# Classification of Pulmonary Nodules by Using Hybrid Features

**DOI:** 10.1155/2013/148363

**Published:** 2013-06-25

**Authors:** Ahmet Tartar, Niyazi Kilic, Aydin Akan

**Affiliations:** ^1^Department of Engineering Sciences, Istanbul University, 34320 Avcılar, Istanbul, Turkey; ^2^Department of Electrical and Electronics Engineering, Istanbul University, 34320 Avcılar, Istanbul, Turkey

## Abstract

Early detection of pulmonary nodules is extremely important for the diagnosis and treatment of lung cancer. In this study, a new classification approach for pulmonary nodules from CT imagery is presented by using hybrid features. Four different methods are introduced for the proposed system. The overall detection performance is evaluated using various classifiers. The results are compared to similar techniques in the literature by using standard measures. The proposed approach with the hybrid features results in 90.7% classification accuracy (89.6% sensitivity and 87.5% specificity).

## 1. Introduction

 Computer aided detection (CAD) system is an extremely important task for the detection of pulmonary nodules in medical images. To attain a more reliable and accurate diagnosis, CAD systems have been recently developed to assist interpretation of the medical images. The systems that find true positive findings from the medical images are especially important in that they can also help radiologists in the identification of early stage pulmonary nodules. To best interpret the information revealed in the images, experienced physicians are required; however, such experts may reach different diagnosis results for the same set of medical imaging. Thus, CAD system is an intensive tool that can provide radiologists with a second opinion to improve the sensitivity of their diagnosis decision-making process [1]. The aim of a CAD system is to provide diagnosis information to improve clinical decision-making process; therefore, its success is related directly to its disease detection accuracy [2]. Today, CAD systems are frequently utilized to detect and diagnose numerous abnormalities in routine clinical work. CAD systems are usually specialized in anatomical regions such as the thorax, breast, or colon by using certain medical imaging technologies such as radiography, computed tomography (CT), or magnetic resonance imaging (MRI) [3].

 Recently, lung cancer is still considered a major cause of deaths from cancer worldwide. In particular, it is one of the main public health issues in the developed industrial countries [4, 5]. This makes the treatment of lung cancer a very important task in the war against cancer. Early detection of potentially cancerous pulmonary nodules is highly important for improving the patient's chance of survival. Multidetector computed tomography system is a very sensitive imaging modality to detect small pulmonary nodules.

In previous studies, classification systems were developed by using the features of nodule candidate patterns with image-processing techniques [6–8], by classifying the shape of pulmonary nodule patterns [9, 10] and by using morphological features [11, 12]. To classify lung nodules, neural network approaches  [13, 14] and Fisher linear discriminant classifier [15, 16] were proposed. In addition, several approaches have been proposed to detect pulmonary nodules in thin-slice helical computed tomography images [17, 18]. Similar techniques are introduced by using genetic algorithm with the random subspace method [19, 20], a single support vector machine [21], and random forest classifiers [22, 23]. Recently, the ensemble learning methods have been applied to classification problems [24, 25]. Especially, the ensemble learning algorithms such as bagging and adaboost are shown to be superior to a single classifier [26, 27].

 In this study, a combination of four different methods was proposed for feature extraction from CT images.

*Method 1*. Two-dimensional principal component analysis (2D-PCA) applied to dataset.

*Method 2.* Statistical features obtained from 2D-PCA values. 

*Method 3*. Geometric features obtained by using the regional descriptors of the 2D patterns based on the basic morphological shape information.

*Method 4*. Selecting the best features of the above three methods with mRMR (minimum Redundancy Maximum Relevance) method, hybrid features are obtained by combining the best features.

 To perform a rigorous validation with the proposed system, completely independent training and testing datasets are utilized. All nodules in the dataset are first tuned/trained using a dataset provided as a courtesy of the University of Istanbul, Cerrahpasa Faculty of Medicine.

 A classification task forms the backbone of a computer aided detection system. In this paper, we propose a new classification approach for pulmonary nodules using hybrid features to be used in such a CAD system. The objective of the proposed study is to analyze the effect of the hybrid features on classification of pulmonary nodules. The proposed classification approach has several novel potential roles.

- To be used as an effective filtering method to reduce the number of false positives in a CAD system.
- To increase the diagnostic accuracy of the detection system.

The rest of this paper is organized as follows. The proposed classification approach for a CAD system and methods used in the algorithm are described in Section 2. This section includes the database information, feature extraction, feature selection, and classifier algorithms. Overall performance of the proposed system as well as comparisons with six other previously presented CAD systems is presented in Section 3. Conclusions are given in Section 4.

## 2. Materials and Methods

### 2.1. Pulmonary Nodule Database and Imaging Protocol

**Figure 1:**
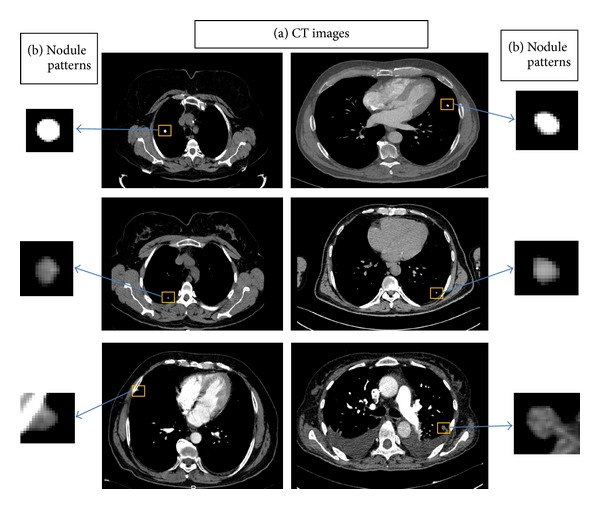
Two-dimensional image samples: (a) CT images and (b) pulmonary nodule patterns.

**Figure 2:**
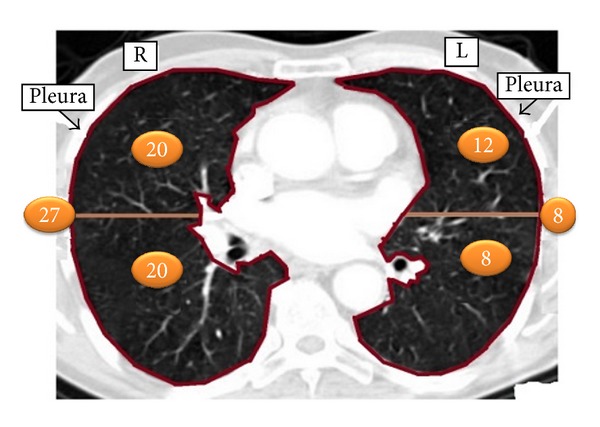
Representation of the number of pulmonary nodules in the right and left lung parenchyma.

In the study, dataset containing 95 pulmonary nodules and 75 nonnodules patterns obtained from two-dimensional (2D) CT images from 63 patients was utilized. The 2D pulmonary nodule patterns are manually marked on CT image by radiologists. Then, the nodule pattern is extracted from the CT image as illustrated in Figure 1. Other patterns in the lung parenchyma similar to nodules but not marked as “nodule” by the radiologists are selected as the member patterns of nonnodule class. Images are collected from 39 male and 24 female patients whose ages are ranging from 25 to 78 years [mean = 55.4 ± 12.3 years]. The number of pulmonary nodules detected in the right and left lung parenchyma, as illustrated in Figure 2, is a total of 67 (20 in the upper part, 20 on the bottom part, and 27 pleural cases) and a total of 28 (12 in the upper part, 8 on the bottom part, 8 pleural case), respectively.

**Figure 3:**
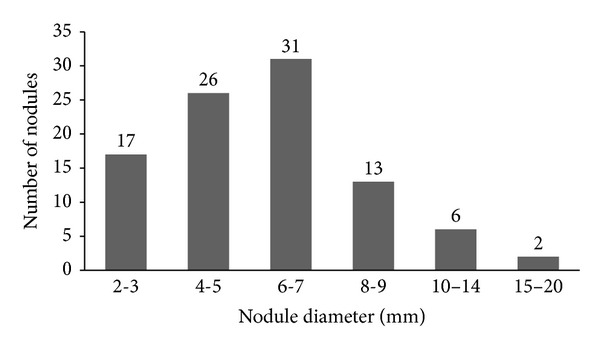
Histogram representing the distribution of the diameter for the 67 nodules of the database.

**Figure 4:**
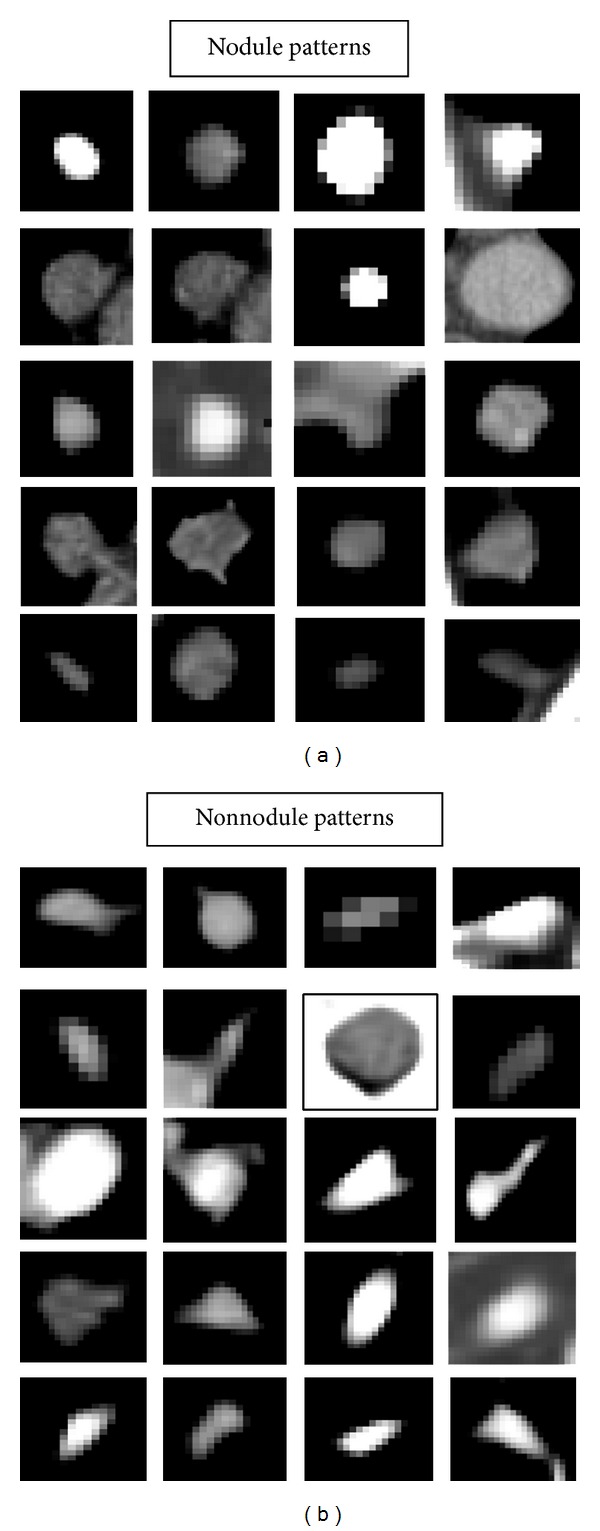
Nodule and nonnodule pattern samples used in dataset.

**Figure 5:**
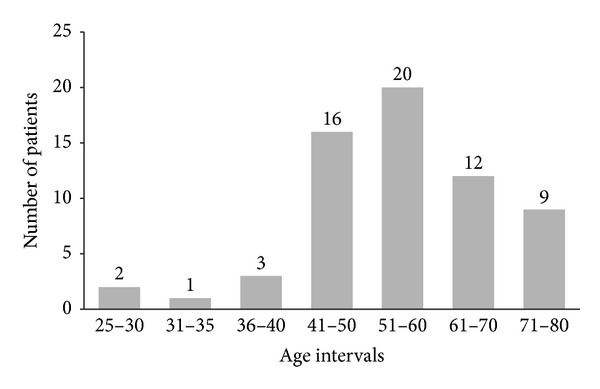
Histogram representing the age distribution of patients.

The average nodule diameter is 6.42 ± 3.00 mm. The diameter distribution of the nodules used in the database is shown in Figure 3. Also nodule and nonnodule pattern samples used in dataset are given in Figure 4. The age distribution of the patients is illustrated in Figure 5.

The dataset was obtained from chest CT images of patients scanned by using “Sensation 16” CT scanner (Siemens Medical Systems) between 2010 and 2012 at Radiology Department, Cerrahpasa Medicine Faculty, Istanbul University. CT scans were acquired at a tube potential voltage of 120 kVp. All CT images are in size of 512 × 512 pixels and stored as DICOM (Digital Imaging and Communications in Medicine) format files, directly from the CT modality.

### 2.2. Feature Extraction

#### 2.2.1. Two-Dimensional Principal Component Analysis* (2D-PCA)*

Principal component analysis (PCA) is defined as a classical dimension reduction method for feature extraction and data representation technique widely used in the areas of pattern recognition, computer vision, and signal processing [28]. Eigenvalue and eigenvector components are ranked according to their variance to the principal axes and ranked from having the most contribution to the least one. Number of the reduced dimension is based on summed contribution of the eigenvalues exceeding 99%. It provides a dimensionality reduction with an unsupervised learning algorithm [29]. Consider the following.

 Let *x* be an *n*-dimensional column vector. The project image *A* is an *mxn* matrix, onto *x* by *y* = *Ax*. In order to determine the optimal projection vector *x*, the total scatter of the projected samples *S*_*x*_ is utilized to measure the optimality of *x*

(1) $$\begin{array}{c} S_{x}=x^{T}E\left\{{\left[A-E\left(A\right)\right]}^{T}\left[A-E\left(A\right)\right]\right\}x=x^{T}S_{A}x, \end{array}$$

where *S*_*A*_ depicts the image covariance matrix.

Suppose that there are *M* training samples *A*_*i*_  {*i* = 1,2, 3,…, *M*} and $\overset{-}{A}$ is the average image,

(2) $$\begin{array}{c} S_{A}=\frac{1}{M}\sum_{i=1}^{M}{\left[A_{i}-\overset{-}{A}\right]}^{T}\left[A_{i}-\overset{-}{A}\right]. \end{array}$$

The optimal projection direction *x*_opt_ denotes the eigenvector of *S*_*A*_ corresponding to the largest eigenvalue. Usually a set of orthonormal projection directions, *x*_1_, *x*_2_,…, *x*_*d*_, are chosen. These projection directions are the orthonormal eigenvectors of *S*_*A*_ corresponding to the first *d* largest eigenvalues.

For a given *A*, let *y*_*k*_ = *Ax*_*k*_ {*k* = 1,2,…, *d*}. A set of projected feature vector *y*_*k*_ and the principal components of *A* are found. The feature matrix of *A* is obtained as *B* = [*y*_1_, *y*_2_,…, *y*_*d*_]. The nearest neighborhood classifier is adopted for classification. The distance between two arbitrary feature matrices, *B*_*i*_ and *B*_*j*_, is given by

(3) $$\begin{array}{c} d\left(B_{i},B_{j}\right)=\sum_{k=1}^{d}{||y_{k}^{i}-y_{k}^{j}||}_{2}, \end{array}$$

where ||*y*_*k*_^*i*^−*y*_*k*_^*j*^||_2_ depicts the Euclidean distance between *y*_*k*_^*i*^ and *y*_*k*_^*j*^ [30].

**Figure 6:**
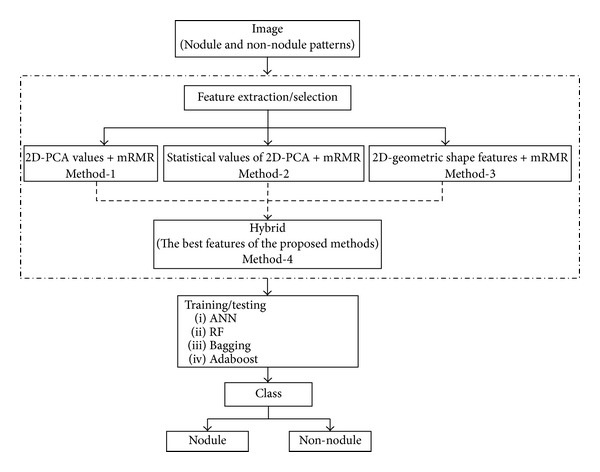
The classification scheme proposed for a CAD system.

A classification process is the basis of a computer aided detection system. The classification scheme proposed for a computer-aided detection algorithm used in this work is shown in Figure 6.

#### 2.2.2. Morphological Image Processing

 Morphology is a cornerstone of the mathematical set of tools underlying the development of techniques that extract the meaning features from an image [31]. To extract the features of pulmonary nodules, geometric features were obtained by using the regional descriptors of the 2D patterns based on the basic morphological shape information. The geometric features consist of the area, perimeter, diameter, solidity, eccentricity, aspect ratio, compactness, roundness, circularity, ellipticity of the patterns in this study.

 These features are given by its definitions in Table 1. A total of 10 features are evaluated for extracting features of the patterns. From these features, *Solidity* denotes the proportion of the pixels in the convex hull that are also in the region. *Eccentricity* depicts the eccentricity of the ellipse that has the same second moments as the region. Also it is the ratio of the distance between the foci of the ellipse and its major axis length. The value of eccentricity is between 0 and 1. Measurements of compactness, roundness, circularity and ellipticity are computed by the definitions given in Table 1 [37].

### 2.3. Feature Selection

#### 2.3.1. The mRMR Method

 The mRMR (minimum Redundancy Maximum Relevance) method from the feature selection methods has been providing shorter calculation time and higher accuracy for the classifier. The mRMR method was proposed by Peng et al. [38]. The mRMR method uses the mutual information between a feature and a class or a feature and another feature. The relevance of a feature set *S* for the class *c* is defined by the average of all mutual information values between individual feature *x*_*i*_ and class *c*,

(4) $$\begin{array}{c} \max D\left(S,c\right),\;D=\frac{1}{\left|S\right|}\sum_{x_{i}\in S}I\left(x_{i};c\right), \end{array}$$

where *I*(*x*_*i*_, *c*) denotes the mutual information between feature *x*_*i*_ and class *c*. The redundancy of all features in the set *S* is defined by the average of all mutual information values between the feature *x*_*i*_ and the feature *x*_*j*_,

(5) $$\begin{array}{c} \min R\left(S\right),\;R=\frac{1}{{\left|S\right|}^{2}}\sum_{x_{i}x_{j}\in S}I\left(x_{i},x_{j}\right), \end{array}$$

where *I*(*x*_*i*_, *x*_*j*_) is the mutual information between features *x*_*i*_ and *x*_*j*_. The mRMR criteria, that is, the combination of two measures given in (4) and (5), are given by the following terms:

(6) $$\begin{array}{c} \text{the}\;\text{difference}\;\text{criterion:}\;\;\max\left(D-R\right), \end{array}$$

(7) $$\begin{array}{c} \text{the}\;\text{quotient}\;\text{criterion:}\;\;\max\left(\frac{D}{R}\right). \end{array}$$

As a result, the best feature set is obtained by optimizing expressions of (4) and (5) simultaneously according to (6) or (7).

### 2.4. Nodule Classification

#### 2.4.1. Artificial Neural Network

**Figure 7:**
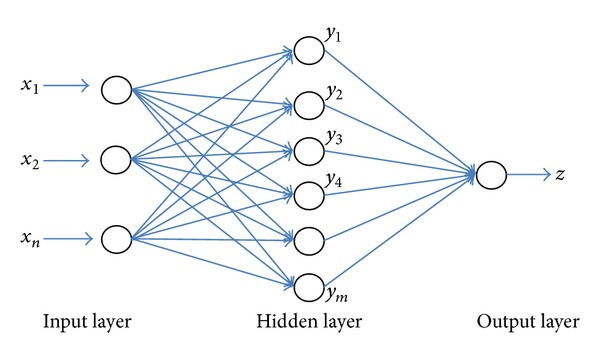
An artificial neural network structure.

An artificial neural network (ANN) is one of the tools of artificial intelligence intended to imitate the complex operation of organizing and processing information of the neurons in the human brain. ANN can recognize patterns correlating strongly with a set of data which correspond to a class by a learning process, in which interneuron connection weights are utilized to store knowledge about specific features identified within the data [39]. It is used for reducing experimental work and time losses. A common ANN is the multilayer perceptron (MLP) algorithm which is made up from three layers as shown in Figure 7. The ANN is trained by entering information from the input layer through the hidden and output layers of the network [40]. The ANN is performed by using the back-propagation algorithm based on the Levenberg-Marquardt rule [41].

The output signal for the *l*th neuron in the *n*th layer is given by the following expression:

(8) $$\begin{array}{c} y_{l}^{n}\left(t\right)=\varphi \left[\sum_{j=1}^{p}w_{lj}^{n}\left(t\right)y_{j}^{n-1}\left(t\right)+\Psi_{l}^{n}\right], \end{array}$$

where *φ*(·) denotes the activation function, *w*_*lj*_^*n*^ depicts the connection weight, *t* denotes the time index, and Ψ_*l*_^*n*^ = *w*_*l*_*o*__^*n*^(*t*) depicts the weights. The synaptic weight *w*_*ji*_^*n*^(*t*) is defined by the following expression (1 ≤ *n* ≤ *N*):

(9) $$\begin{array}{c} {\Delta w}_{ji}^{n}\left(t+1\right)=w_{ji}^{n}\left(t\right){+\Delta w}_{ji}^{n}\left(t\right). \end{array}$$

And it is revised as the following:

(10) $$\begin{array}{c} {\Delta w}_{ji}^{n}\left(t\right)={\eta \lambda }_{j}^{n}\left(t\right)y_{i}^{n-1}\left(t\right), \end{array}$$

where *η* depicts the learning rate (0 < *η* < 1). Also the local error gradient is given by

(11) $$\begin{array}{c} \lambda_{j}^{n}\left(t\right)\equiv \frac{{\partial E}_{t}}{{\partial u}_{j}^{n}}. \end{array}$$

To improve the performance of the back-propagation algorithm, a momentum term *α* is added as the following:

(12) $$\begin{array}{c} {\Delta w}_{ji}^{n}\left(t\right)={\eta \lambda }_{j}^{n}\left(t\right)y_{i}^{n-1}\left(t\right)+{\alpha \Delta w}_{ji}^{n}\left(t-1\right), \end{array}$$

where *α* is between 0 and 1. For the output layer, the local error gradient is defined by

(13) $$\begin{array}{c} \lambda_{j}^{N}\left(t\right)=\left[d_{j}\left(t\right)-y_{j}^{N}\left(t\right)\right]\varphi \left[u_{j}^{N}\left(t\right)\right]\equiv e_{j}\left(t\right)\varphi \left[u_{j}^{N}\left(t\right)\right], \end{array}$$

where *d*_*j*_(*t*), *φ*(·) depict the goal output signal and the activation function, respectively.

#### 2.4.2. Random Forest

Random forest was proposed by Breiman in 1999 [42]. It is a new development in tree based classifiers and fast proven to be one of the most important algorithms in the machine learning. It is defined as a combination of tree predictors depending on the values of a random vector sampled independently and with the same distribution for all trees in the forest. Random forest has given robust and improved results of classifications on standard data sets. It is providing very good competition to neural networks and ensemble techniques on different classification problems. Random forest is related to be special type of ensembles using bagging and random splitting methods to grow multiple trees [42, 43].

There are several advantages for the Random forest method. Especially, Random forest can predict what features are important in the classification. It can process efficiently large data sets. Also it can be utilized as an effective method to estimate missing data.

#### 2.4.3. Bagging

 Bagging is unstable learning algorithm for small data set if small changes in the training data will generate very diverse classifiers. The use of bagging to improve performance by taking advantage of this effect was proposed by Breiman [44]. A single classifier could have a higher test error. The combination of classifiers can produce a lower test error than that of the single classifier because the diversity of classifiers usually compensates for errors of any single classifier [45]. A learning algorithm combination in those small changes in the training set leads to relatively large changes in accuracy.

#### 2.4.4. Adaboost

 Adaboost is one of the powerful methods for pattern recognition [46]. Adaboost classifier firstly introduced by Freund and Schapire [47, 48] is an ensemble classifier composed of many weak classifiers for the two-class classification problem. It generates strong classifier with weak classifiers. Adaboost makes a committee of member weak classifiers by adaptively adjusting the weights at each loop. While the weights of the training patterns classified correctly by a weak classifier are decreased, the weights of the training patterns misclassified by the weak classifier are increased.

 Adaboost algorithm shows good performance effect because of the ability to generate expanding diversity. In order to improve the performance result of the final ensemble, adaboost algorithms consist of diverse weak classifiers. Especially, the boosting algorithm adaboost.M1—the first directly—extends the original adaboost algorithm to the multiclass case without reducing it to multiple two-class problems. 

 Principal component analysis, mRMR method, and morphological image processing algorithms are performed by using the Matlab codes. Classification processes were provided by using data mining software called the Weka tool version 3.7.7 which is available from http://www.cs.waikato.ac.nz/~ml/weka/. Tests are done on a PC with Intel Core i7, 1.90 GHz CPU, and 4.00 GB RAM. For evaluating the classifiers, 5-fold cross-validation technique is used.

## 3. Results

 Various classification methods are utilized for feature extraction and selection in medical pattern recognition. In this study, two-dimensional principal component analysis and geometric feature values were used for feature extraction. The mRMR method was applied for feature selection. The entire dataset is randomly partitioned into training and testing sets. The entire dataset is divided into approximately 50% training dataset and 50% test dataset. The training dataset consists of 47 pulmonary nodules and 37 nonnodule patterns (total number of patterns is 84). The test dataset consists of 48 pulmonary nodules and 38 nonnodule patterns (a total of 86 patterns). The best features for each method are determined using the mRMR feature selection only in the training dataset. Then, the classification accuracies of the methods are calculated using these features in the test dataset.

In the study, four different methods were proposed. For principal component analysis on method 1, the largest first seven values were selected for the first seven principal components because of highest variance value. So that, a 7 × 7-dimensional matrix was formed for each pattern. Then, 1 × 49-dimensional feature vector was obtained. In this way, at least 99% value of the total variance for each pattern was taken into account. To select the best features that contribute to the performance of classification system in the training set, the mRMR method was utilized. The number of best features performed with the mRMR method was determined as 20.

 In method 2, the statistical features, *minimum *(min)*, maximum *(max)*, mean, standard deviation* (std), *variance* (var), and 3rd *moment *values, are calculated in the training dataset. Thus, a 1 × 6-dimensional feature vector was obtained. The best feature ranking that performed with the mRMR method is 3rd *moment, min, mean, std, max, and var.* The number of best features performed with the mRMR method was the first 5 features which are 3rd* moment, min, mean, std, *and *max*.

 In method 3, geometric features based on the basic morphological shape information were utilized for the 2D patterns in the training dataset. The geometric features include the area, perimeter, diameter, solidity, eccentricity, aspect ratio, compactness, roundness, circularity, and ellipticity of the patterns. The number of best features performed with the mRMR method was 5 features consisting of *compactness, aspect ratio, area, solidity* and *ellipticity*.

 A new hybrid approach for classification was introduced on method 4. A new feature vector was created by combining the best features of the above three methods, aiming at increasing the sensitivity of the proposed classification approach. A total of 30 features selected by the three methods were now applied to the test dataset.

 Random forest, artificial neural networks, ensemble bagging with RF, ensemble bagging with ANN, ensemble adaboost with RF, and ensemble adaboost with ANN classifiers were separately applied in all of the methods.

 The classifiers were compared, and overall performance results of the proposed classification approach were given in Table 2. The performance measurements are given by

(14) $$\begin{array}{c} \text{sensitivity}=\frac{\text{TP}}{\text{TP}+\text{FN}}, \end{array}$$

(15) $$\begin{array}{c} \text{specificity}=\frac{\text{TN}}{\text{TN}+\text{FP}}, \end{array}$$

(16) $$\begin{array}{c} \text{TCA}=\frac{\text{TP}+\text{TN}}{\text{TP}+\text{FP}+\text{TN}+\text{FN}}, \end{array}$$

(17) $$\begin{array}{c} \text{RMSE}=\sqrt{\frac{\sum {\left(y^{\prime }-y\right)}^{2}}{n}}, \end{array}$$

(18) $$\begin{array}{c} \text{FPR}=\frac{\text{FP}}{\text{FP}+\text{TP}}, \end{array}$$

where TP, TN, FP, and FN denote the number of nodules classified as true positive, true negative, false positive, and false negative, respectively. FPR is false positive rate per image.

Sensitivity is the number of correctly predicted positives divided by the total number of positive cases. Specificity is the number of correctly predicted negatives divided by the total number of negative cases. TCA (*total classification accuracy*) represents the probability of correctly classified patterns. For RMSE (*root mean squared error*), *y*, *y*′, and *n* depict actual value, predicted value, and number of data patterns, respectively. In order to measure the performance of the classification system, AUROC is often used as well as sensitivity and specificity [49]. AUROC represents the area under the receiver operating characteristic curve. Kappa statistics is a chance-corrected measure of agreement between the classifications and the true classes. If Kappa is equal to 1, it indicates perfect agreement. If Kappa is equal to 0, it represents chance agreement.

Confusion matrixes of the classifiers in the proposed methods were shown in Table 3.

 A ROC curve is usually used as a technique to visualize the performance of classifiers and is extremely useful to compare the performance of different classifiers in medical decision-making systems. The curve indicates the tradeoff between the true positive and false positive rates.

**Figure 8:**
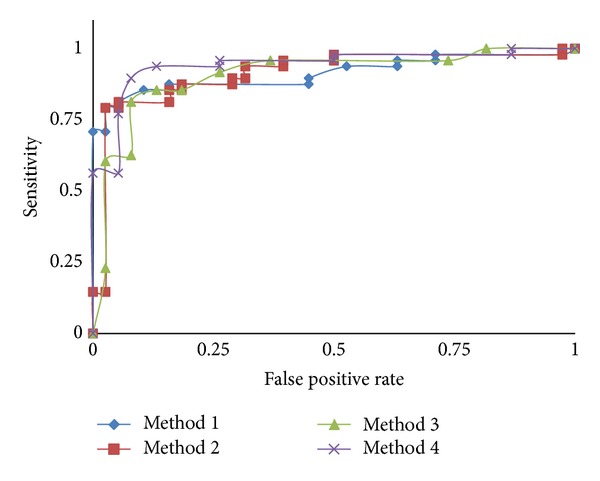
ROC curves showing CAD performance with the methods proposed.

The area under ROC (*AUROC*) used here is largely adopted to represent the expected performance of a classifier. The AUROC of a classifier is equivalent to the probability that the classifier ranks a randomly chosen positive instance higher than a randomly chosen negative instance [50]. For our proposed methods, ROC curves are illustrated in Figure 8.

### 3.1. Performance Comparison

 To evaluate the performance of the classification approach, the results of this study were compared with previously reported CAD systems. It is highly difficult task to make comparison between previously published CAD systems due to different datasets, nodule size or type, and nodule or nonnodule patterns. It is still important to make a relative comparison. It is obviously shown that the performance results of a CAD system can differ significantly depending on those variables.

 A single 2D slice is selected for each 3D object as seen in Figure 1. Pulmonary nodules are observed on a several slice range of the whole CT scan. Radiologists inspect these slices for the 2D patterns then select and label the pulmonary nodule pattern which has the largest dimension (i.e., area, diameter). Thus, when any physician detects a pulmonary nodule on the CT slices, he/she chooses the largest 2D pattern which is labeled and used in the dataset.

 For comparative analysis, it is examined recently and reported that CAD systems have utilized the LIDC (Lung Image Database Consortium) database to evaluate detection systems [32–34]. Opfer and Wiemker [32] utilized the dataset comprised of 93 cases (2-3 mm slice thickness) with 127 nodules. Sahiner et al. used the dataset having a total of 73 nodules by combining 28 CT scans from the LIDC and 20 scans from another database [34]. Rubin et al. used a total of 84 CT scans with a total of 143 nodules in the range of 3–30 mm in nodule size [33]. Other papers utilized their own databases for the performance analysis of CAD system [35, 36, 51]. Suzuki et al. used the dataset of 20 CT scans (1.25 mm slice thickness and 0.6 mm pixel interval) containing 195 noncalcified nodule patterns (≥3 mm) [35]. Tartar et al. utilized low-dose CT images scanned from 71 different patients with a total of 121 nodules (8–20 mm nodule size interval), totaling 101 CT scans (10 mm slice thickness and 0.586–0.684 pixel interval) [36]. Shiraishi et al. used the dataset containing 67 pulmonary nodules and 67 nonnodules obtained from 46 patients in our previous study [51].

 In this study, a dataset containing 95 pulmonary nodules and 75 nonnodules patterns obtained from two-dimensional CT images from 63 patients is used. All of our CT scans are scanned by using the standard imagery protocol. A comparison of the performance of reported CAD systems was shown in Table 4. As seen from the table, the proposed classification approach achieved a sensitivity of 89.6% and an accuracy of 90.7% in the range of 2–20 mm nodule size. All other CAD systems have reasonable sensitivity values in classification of pulmonary nodules. It is extremely important to consider the small nodule size in the classification of a CAD system. This increases the probability of early detection of nodules. Considering these results, it can be seen that the proposed study represents a relatively high sensitivity. In addition, the overall false positive rate per image is calculated as 0.079 by using the expression of (18) for the hybrid approach.

## 4. Conclusions

 In this paper, a new classification approach of pulmonary nodules for a CAD system from CT imagery is presented. An important feature of a CAD system desired by radiologists is that it is able to detect and classify small nodule patterns. The dataset in our study is composed of nodules with relatively smaller diameters (>2 mm), as shown in Figure 3 and Table 4. 

 In the literature, various classification algorithms for CAD systems have been extensively studied. In order to reduce the complexity of the algorithm and the computational load, the use of fewer features is extremely important, while maintaining an acceptable detection performance. For example, the CAD system in Messay et al. [15] uses 40 features selected from a set of 245 features with sensitivity of 82.66%, Hardie et al. [16] uses a subset of 46 features selected from a set of 114 features by sensitivity of 78.1%, and Shiraishi et al. [51] utilizes 71 features by sensitivity of 70.4%, respectively. In this study, in order to choose the best set of image features characterizing the patterns, various feature extraction/selection methods such as 2D-PCA, statistical features of 2D-PCA, morphological image processing based on geometric features, and mRMR feature selection method were implemented. 

 The performances of the proposed approaches are evaluated by using different classifiers and performance metrics such as accuracy, sensitivity, specificity, AUROC, Kappa statistic, and RMSE. The proposed classification approach utilizes 30 features combined by the hybrid approach with sensitivity of 89.6%, accuracy of 90.7, and specificity of 87.5%.

 Considering the test results in Table 2, ensemble learning algorithms yield the best performances on the features suggested in methods 1 and 3. However, especially, in the hybrid approach (method 4) combining the best features of the three methods, nonlinear multilayered ANN is shown to be superior to the other classifiers. Our approach uses ANN classifier with fewer features to avoid generalization problems, high complexity, and computational burden that can be caused by using an ANN with very large number of (potentially irrelevant) features. In addition, as shown in Table 3, false positive (FP) rate is shown to decline in the hybrid approach which provides higher detection performance by using fewer features.

## Figures and Tables

**Table 1:** Geometric features used for pulmonary nodule detection.

Measure	Definition
Area	*A*
Perimeter	*P*
Diameter	*D*
Solidity	*S*
Eccentricity	*E*
Aspect ratio	$\frac{\mathrm{Min. diameter (M)}}{\mathrm{Max. diameter (L)}}$
Compactness	$\frac{P^{2}}{4\pi A}$
Roundness	$\frac{4A}{\pi L^{2}}$
Circularity	$\frac{4\pi A}{P^{2}}$
Ellipticity	$\frac{\pi L^{2}}{2A}$

**Table 2:** Overall performance results of the proposed classification approach.

	Sensitivity (%)	TCA (%)	Specificity (%)	AUROC	Kappa	RMSE
Method 1						
ANN	87.5	84.9	83.8	0.928	0.693	0.356
RF	87.5	80.2	81.8	0.889	0.594	0.375
Bagging (ANN)	100	61.6	100	0.599	0.145	0.557
Bagging (RF)	**87.5**	**86.0**	**84.2**	**0.911**	**0.717**	**0.339**
Adaboost (ANN)	87.5	81.4	82.4	0.920	0.619	0.416
Adaboost (RF)	83.3	75.6	75.8	0.880	0.498	0.384

Method 2						
ANN	**85.4**	**83.7**	**81.6**	**0.913**	**0.670**	**0.327**
RF	83.3	83.7	80.0	0.890	0.672	0.356
Bagging (ANN)	100	60.5	100	0.616	0.116	0.522
Bagging (RF)	83.3	81.4	78.9	0.912	0.623	0.346
Adaboost (ANN)	81.3	80.2	76.9	0.869	0.600	0.385
Adaboost (RF)	83.3	80.2	78.4	0.901	0.598	0.351

Method 3						
ANN	83.3	83.7	80.0	0.893	0.672	0.358
RF	81.3	77.9	75.7	0.869	0.551	0.383
Bagging (ANN)	39.6	59.3	52.5	0.738	0.224	0.482
Bagging (RF)	83.3	84.9	80.5	0.912	0.696	0.344
Adaboost (ANN)	83.3	83.7	80.0	0.867	0.672	0.378
Adaboost (RF)	**85.4**	**83.7**	**81.6**	**0.906**	**0.670**	**0.348**

Method 4						
ANN	**89.6**	**90.7**	**87.5**	**0.940**	**0.812**	**0.307**
RF	85.4	83.7	81.6	0.908	0.670	0.335
Bagging (ANN)	60.4	66.3	59.6	0.770	0.333	0.463
Bagging (RF)	87.5	86.0	84.2	0.922	0.717	0.325
Adaboost (ANN)	87.5	86.0	84.2	0.921	0.717	0.334
Adaboost (RF)	85.4	80.2	80.0	0.911	0.596	0.343

**Table 3:** Confusion matrixes classified by the proposed methods.

	TP	FP	FN	TN
Method 1				
ANN	42	6	7	31
RF	42	6	11	27
Bagging (ANN)	48	0	33	5
Bagging (RF)	**42**	**6**	**6**	**32**
Adaboost (ANN)	42	6	10	28
Adaboost (RF)	40	8	13	25

Method 2				
ANN	**41**	**7**	**7**	**31**
RF	40	8	6	32
Bagging (ANN)	48	0	34	4
Bagging (RF)	40	8	8	30
Adaboost (ANN)	39	9	8	30
Adaboost (RF)	40	8	9	29

Method 3				
ANN	40	8	6	32
RF	39	9	10	28
Bagging (ANN)	19	29	6	32
Bagging (RF)	40	8	5	33
Adaboost (ANN)	40	8	6	32
Adaboost (RF)	**41**	**7**	**7**	**31**

Method 4				
ANN	**43**	**5**	**3**	**35**
RF	41	7	7	31
Bagging (ANN)	29	19	10	28
Bagging (RF)	42	6	6	32
Adaboost (ANN)	42	6	6	32
Adaboost (RF)	41	7	10	28

**Table 4:** Comparison of the classification performance of reported CAD systems.

CAD system	Nodule size used (mm)	Reported sensitivity (%)
Opfer and Wiemker [32]	≥4	74.0
Rubin et al. [33]	≥3	76.0
Sahiner et al. [34]	3–36.4	79.0
Suzuki et al. [35]	8–20	80.3
Tartar et al. [36]	2–20	82.1
Messay et al. [15]	3–30	82.66
Proposed approach	**2–20**	**89.6**

## References

[1] Doi K (2007). Computer-aided diagnosis in medical imaging: historical review, current status and future potential. *Computerized Medical Imaging and Graphics*.

[2] Giger ML, Chan H-P, Boone J (2008). Anniversary paper: history and status of CAD and quantitative image analysis: the role of Medical Physics and AAPM. *Medical Physics*.

[3] Summers RM (2003). Road maps for advancement of radiologic computer-aided detection in the 21st century. *Radiology*.

[4] Cancer facts and figs.

[5] Jemal A, Siegel R, Ward E, Hao Y, Xu J, Thun MJ (2009). Cancer statistics, 2009. *CA Cancer Journal for Clinicians*.

[6] Kanazawa K, Kawata Y, Niki N (1998). Computer-aided diagnosis for pulmonary nodules based on helical CT images. *Computerized Medical Imaging and Graphics*.

[7] Baĝci U, Bray M, Caban J, Yao J, Mollura DJ (2012). Computer-assisted detection of infectious lung diseases: a review. *Computerized Medical Imaging and Graphics*.

[8] Murphy K, van Ginneken B, Schilham AMR, de Hoop BJ, Gietema HA, Prokop M (2009). A large-scale evaluation of automatic pulmonary nodule detection in chest CT using local image features and k-nearest-neighbour classification. *Medical Image Analysis*.

[9] Iwano S, Nakamura T, Kamioka Y, Ishigaki T (2005). Computer-aided diagnosis: a shape classification of pulmonary nodules imaged by high-resolution CT. *Computerized Medical Imaging and Graphics*.

[10] Iwano S, Nakamura T, Kamioka Y, Ikeda M, Ishigaki T (2008). Computer-aided differentiation of malignant from benign solitary pulmonary nodules imaged by high-resolution CT. *Computerized Medical Imaging and Graphics*.

[11] Chen H, Zhang J, Xu Y, Chen B, Zhang K (2012). Performance comparison of artificial neural network and logistic regression model for differentiating lung nodules on CT scans. *Expert Systems with Applications*.

[12] Kubota T, Jerebko AK, Dewan M, Salganicoff M, Krishnan A (2011). Segmentation of pulmonary nodules of various densities with morphological approaches and convexity models. *Medical Image Analysis*.

[13] Lin D-T, Yan C-R, Chen W-T (2005). Autonomous detection of pulmonary nodules on CT images with a neural network-based fuzzy system. *Computerized Medical Imaging and Graphics*.

[14] Retico A, Delogu P, Fantacci ME, Gori I, Preite Martinez A (2008). Lung nodule detection in low-dose and thin-slice computed tomography. *Computers in Biology and Medicine*.

[15] Messay T, Hardie RC, Rogers SK (2010). A new computationally efficient CAD system for pulmonary nodule detection in CT imagery. *Medical Image Analysis*.

[16] Hardie RC, Rogers SK, Wilson T, Rogers A (2008). Performance analysis of a new computer aided detection system for identifying lung nodules on chest radiographs. *Medical Image Analysis*.

[17] Suárez-Cuenca JJ, Tahoces PG, Souto M (2009). Application of the iris filter for automatic detection of pulmonary nodules on computed tomography images. *Computers in Biology and Medicine*.

[18] Hanamiya M, Aoki T, Yamashita Y, Kawanami S, Korogi Y (2012). Frequency and significance of pulmonary nodules on thin-section CT in patients with extrapulmonary malignant neoplasms. *European Journal of Radiology*.

[19] Lee MC, Boroczky L, Sungur-Stasik K (2010). Computer-aided diagnosis of pulmonary nodules using a two-step approach for feature selection and classifier ensemble construction. *Artificial Intelligence in Medicine*.

[20] Choi W-J, Choi T-S (2012). Genetic programming-based feature transform and classification for the automatic detection of pulmonary nodules on computed tomography images. *Information Sciences*.

[21] Wang Q, Kang W, Wu C, Wang B (2013). Computer-aided detection of lung nodules by SVM based on 3D matrix patterns. *Clinical Imaging*.

[22] Lee SLA, Kouzani AZ, Hu EJ (2010). Random forest based lung nodule classification aided by clustering. *Computerized Medical Imaging and Graphics*.

[23] Bosch A, Zisserman A, Muñoz X Image classification using random forests and ferns.

[24] Bauer E, Kohavi R (1999). An empirical comparison of voting classification algorithms: bagging, boosting, and variants. *Machine Learning*.

[25] Maclin R, Opitz D Empirical evaluation of bagging and boosting.

[26] An T-K, Kim M-H A new diverse AdaBoost classifier.

[27] Zhang Z, Xie X Research on AdaBoost.M1 with random forest.

[28] Jain AK, Duin RPW, Mao J (2000). Statistical pattern recognition: a review. *IEEE Transactions on Pattern Analysis and Machine Intelligence*.

[29] Hargrove LJ, Li G, Englehart KB, Hudgins BS (2009). Principal components analysis preprocessing for improved classification accuracies in pattern-recognition-based myoelectric control. *IEEE Transactions on Biomedical Engineering*.

[30] Kong H, Wang L, Teoh EK, Li X, Wang J-G, Venkateswarlu R (2005). Generalized 2D principal component analysis for face image representation and recognition. *Neural Networks*.

[31] Gonzales R, Woods R (2007). *Image Processing*.

[32] Opfer R, Wiemker R Performance analysis for computer-aided lung nodule detection on LIDC data.

[33] Rubin GD, Lyo JK, Paik DS (2005). Pulmonary nodules on multi-detector row CT scans: performance comparison of radiologists and computer-aided detection. *Radiology*.

[34] Sahiner B, Hadjiiski LM, Chan H-P Effect of CAD on radiologists' detection of lung nodules on thoracic CT scans: observer performance study.

[35] Suzuki K, Armato SG, Li F, Sone S, Doi K (2003). Massive training artificial neural network (MTANN) for reduction of false positives in computerized detection of lung nodules in low-dose computed tomography. *Medical Physics*.

[36] Tartar A, Kılıç N, Akan A Bagging support vector machine approaches for pulmonary nodule detection.

[37] Solomon C, Breckon T (2011). *Fundamentals of Digital Image Processing: A Practical Approach With Examples in Matlab*.

[38] Peng H, Long F, Ding C (2005). Feature selection based on mutual information: criteria of max-dependency, max-relevance, and min-redundancy. *IEEE Transactions on Pattern Analysis and Machine Intelligence*.

[39] Lancashire L, Ugurel S, Creaser C, Schadendorf D, Rees R, Ball G Utilizing artificial neural networks to elucidate serum biomarker patterns which discriminate between clinical stages in melanoma.

[40] Erdal HI, Karakurt O, Namli E (2013). High performance concrete compressive strength forecasting using ensemble models based on discrete wavelet transform. *Engineering Applications of Artificial Intelligence*.

[41] Mukherjee I, Routroy S (2012). Comparing the performance of neural networks developed by using Levenberg-Marquardt and Quasi-Newton with the gradient descent algorithm for modelling a multiple response grinding process. *Expert Systems with Applications*.

[42] Breiman L (1999). Random forests.

[43] Breiman L (2001). Random forests. *Machine Learning*.

[44] Breiman L (1996). Bagging predictors. *Machine Learning*.

[45] Opitz D, Maclin R (1999). Popular ensemble methods: an empirical study. *Journal of Artificial Intelligence Research*.

[46] Fan H, Wang H Preditcing protein subcellular location by AdaBoost.M1 algorithm.

[47] Freund Y, Schapire RE Experiments with a new boosting algorithm.

[48] Freund Y, Schapire RE (1997). A decision-theoretic generalization of on-line learning and an application to boosting. *Journal of Computer and System Sciences*.

[49] Li B, Chen K, Tian L, Yeboah Y, Ou S (2013). Detection of pulmonary nodules in CT images based on fuzzy integrated active contour model and hybrid parametric mixture model. *Computational and Mathematical Methods in Medicine*.

[50] Fawett T (2003). ROC graphs: notes and practical considerations for data mining researches.

[51] Shiraishi J, Li Q, Suzuki K, Engelmann R, Doi K (2006). Computer-aided diagnostic scheme for the detection of lung nodules on chest radiographs: localized search method based on anatomical classification. *Medical Physics*.

